# Factors influencing the success and complications of intraosseous access in pediatric patients—a prospective nationwide surveillance study in Germany

**DOI:** 10.3389/fped.2023.1294322

**Published:** 2023-11-29

**Authors:** Daniel Pfeiffer, Martin Olivieri, Sebastian Brenner, Delphina Gomes, Victoria Lieftüchter, Florian Hoffmann

**Affiliations:** ^1^ Dr. v. Hauner Children’s Hospital, Pediatric Intensive Care Unit, Ludwig-Maximilians-University Munich, Munich, Germany; ^2^ Division of Pediatric Intensive Care, Department of Pediatrics, University Hospital Carl Gustav Carus, Dresden, Germany

**Keywords:** critical care, pediatric emergency care, pediatric intensive care, vascular access, intraosseous access, resuscitation, pediatric

## Abstract

**Background:**

Vascular access is essential for the efficient treatment of critically ill children, but it can be difficult to obtain. Our study was conducted to analyze the feasibility and short-term safety of intraosseous access (IO) use as well as factors influencing its success and the incidence of complications in pediatric emergencies and resuscitation. This dataset of systematically documented intraosseous access attempts constitutes one of the largest published in the literature.

**Methods:**

Two-year nationwide prospective surveillance study in Germany from July 2017 to June 2019. Pediatric hospitals anonymously reported the case data of all children aged 28 days to 18 years who arrived with or were treated with an intraosseous access to the German Pediatric Surveillance Unit (GPSU). The main outcomes were the occurrence of complications, overall success and success at the first attempt. The influence of individual factors on outcomes was evaluated using multivariate regression models.

**Results:**

A total of 417 patients underwent 549 intraosseous access attempts. The overall rates of success and success at the first attempt were 98.3% and 81.9%, respectively. Approximately 63.6% of patients were successfully punctured within 3 min from the time of indication. Approximately 47.7% of IO access attempts required patient resuscitation. Dislocation [OR 17.74 (5.32, 59.15)] and other complications [OR 9.29 (2.65, 32.55)] occurred more frequently in the prehospital environment. A total of 22.7% of patients experienced minor complications, while 2.5% of patients experienced potentially severe complications.

**Conclusion:**

We conclude that intraosseous access is a commonly used method for establishing emergency vascular access in children, being associated with a low (age-dependent) rate of severe complications and providing mostly reliable vascular access despite a relatively high rate of dislocation.

## Introduction

Vascular access is paramount for the efficient treatment of critically ill children. Because multiple attempts to establish intravenous access increase mortality rates due to prolonged on-scene times or delayed application of drugs, choosing the best site for quick and reliable vascular access plays an important role in increasing the chances of survival (1, 2).

Establishing a conventional intravenous line in a critically ill pediatric patient is challenging and fails in almost 1/3 of patients treated in the prehospital environment (3). According to several guidelines, the intraosseous route should be considered in the early stage of life-threatening pediatric emergencies, and, as a result of such recommendation, the intraosseous access has been increasingly used in past decades, especially in the prehospital setting (4–7). As many as 1 in 4 critically ill children arrive at a hospital with an intraosseous cannula (3). However, time constraints associated with pediatric emergencies and anatomic challenges, such as a thickened subcutaneous layer and small target structures, pose challenges even for experienced providers (7).

Although intraosseous access is widely regarded as facilitating fast and reliable vascular access to administer drugs and fluids, there are only a few studies on success rates in children and long- and short-term safety in terms of adverse outcomes. Furthermore, it remains unclear whether findings from adult patient studies can be translated to pediatric patients. Studies with a large sample size have yet to be published (7).

This study aims to evaluate the safety and success rates and influencing factors of the use of intraosseous devices in children being treated in the hospital and the prehospital setting.

## Methods

### Study population and case reporting

The present study includes the clinical data of all pediatric patients who are included in the GPSU database and who received IO access in a clinical or/and an out-of-hospital setting in Germany between their first month of life and adulthood. The data of patients who met the case definition were reported monthly to GPSU, from 1 July 2017 to 30 June 2019. GPSU is a well-established hospital-based nationwide prospective surveillance system for rare pediatric diseases covering 345 pediatric hospitals and departments in Germany. Regular analysis of its capture rates showed that reporting completeness of the GPSU surveillance system consistently exceeds 95% (8).

### Ethical approval and consent to participate

All surveillance studies of the GPSU are based on a common study and data protection protocol. The local ethics committee of the Rhineland-Palatinate Medical Association and the state representative for data protection in Rhineland-Palatinate have approved this protocol. A waiver of written informed consent was granted by the Ethics Committee of the State Medical Association of Rhineland-Palatinate (Number 2020-15400_1) and the state representative for data protection in Rhineland-Palatinate as participants were not subjected to any study-related procedures and data were collected in a completely anonymous form.

All analyzed data involving human participants were collected in accordance with the ethical standards outlined in the 1964 Helsinki declaration and its later amendments or comparable ethical standards. In addition to the general ethical approval of the GPSU, the study was approved by the data protection office and the ethics committee of the medical faculty of the Ludwig-Maximilians-University, Munich Nr. 641-16 (19-12-2016).

### Data collection

In this prospective, population-based surveillance study, all the involved German pediatric hospitals and departments received a monthly reminder to report the data of all patients with IO access, including the data of patients who were punctured in their center and the data of those who were transferred with IO access from another center or prehospital setting, as far as known in the receiving center. The report of a case prompted the dissemination of a study-specific pseudonymized questionnaire that was designed by a consensus panel of pediatric emergency specialists of the German Society for Neonatology and Pediatric Intensive Care Medicine (GNPI) and German Interdisciplinary Society for Intensive Care and Emergency Medicine (DIVI).

The questionnaire was completed by the attending pediatrician and then returned with anonymized patient information to GPSU in Mainz (formerly Düsseldorf), Germany. The questionnaire included details on demographic data (age, sex, gestational age), medical indication for the use of an IO needle and diagnosis, IO access use (number of attempts, anatomical location of needle placement, system used, qualification of attending physician, retrospectively estimated time to first access, alternative vascular access routes attempted, infused/applied substances, complications, and time to removal), and the course of the disease and outcome. The overall response rate to the distributed questionnaires after report of a case was 94.4%. All reports were checked for plausibility of data by 2 pediatric intensivists.

Overall success was defined as a patient who received an IO access that facilitated the application for an appropriate amount of time to administer necessary drugs to treat the patient, regardless of the number of attempts.

### Statistical analysis

Data were entered into a Microsoft Office Access 2003 (Version 11.0) database and transferred to the R statistical package, version 3.6.2, for group comparisons. We compared patient groups by analyzing criteria at the patient level as well as on an individual IO attempt basis using Student's t test, χ^2^ test and Fisher's exact test as appropriate.

## Results

Over the study period of two years, the clinical data of 417 patients who underwent 549 attempts to establish intraosseous access were included in the analysis. More than 1 access attempt was made in 110 patients (26.4%). A total of 87.8% were between 1 month and 6 years old. In 199 patients (47.7%), the indication included ongoing cardiopulmonary resuscitation (CPR).

Table 1 provides an overview of the basic characteristics of the study population.

**Table 1:** Basic characteristics of the study population.

Age structure (*n* = 417)	Male	Female	*n* (%)
1–12 months	101 (58.4)	72 (41.6)	173 (41.5)
1–6 years	106 (54.9)^a^	85 (44.0)	193 (46.3)
6–12 years	15 (50.0)	15 (50.0)	30 (7.2)
>12 years	12 (57.1)	9 (42.9)	21 (5.0)
All	234 (56.1)	181 (43.4)	417 (100.0)
Removal of intraosseous needle (*n* = 307)^b^	*n* (%)	*n* (%)	
<1 h	40 (13.0)	209 (68.0)	
1–6 h	169 (55.0)		
6–12 h	50 (16.3)	86 (28.0)	
12–24 h	36 (11.7)		
>24 h	12 (3.9)	12 (3.9)	
Anatomical location of intraosseous access attempt (*n* = 530)	Individual attempts *n* (%)	Right side *n* (%)	Left side *n* (%)
Tibia	519 (97.9)^c^	265 (51.1)	239 (46.1)
Proximal tibia	509 (96.0)^c^	260 (51.1)	234 (46.0)
Distal tibia	9 (1.7)	4 (44.4)	5 (55.6)
Distal femur	4 (0.8)^c^	2 (50.0)	1 (25.0)
Proximal humerus	6 (1.1)^c^	2 (33.3)	3 (50.0)
Spina iliaca ant. sup.	1 (0.2)^c^	–	–
System used (*n* = 494)	Individual attempts *n* (%)	Of which successful (*n* = 445, 90.1%)	
EZIO semiautomatic drill	460 (93.1)	377 (82.0)	
EZIO drill manual use	5 (1.0)	3 (60.0)	
Cook needle	26 (5.3)	18 (69.2)	
Bone injection gun (BIG)	3 (0.6)	3 (100.0)	
GRH: Time from indication to successful IO placement^e^ (*n* = 226)	Cumulative *n* (%)	Individual *n* (%)	
<1 min	31 (13.7)	31 (13.7)	
<2 min	100 (44.2)	69 (30.5)	
<3 min	144 (63.7)	44 (19.5)	
<4 min	159 (70.4)	15 (6.6)	
<5 min	192 (85.0)	33 (14.6)	
>6 min	226 (100.0)	34 (15.0)	
GRH: Highest qualified doctor present^e^ (*n* = 230)^d^	*n* (%)	Success rate [%] (mean attempts)	
Department head	5 (2.2)	66.7 (1.80)	
Consultant	113 (49.1)	63.7 (1.51)	
Specialist	63 (27.4)	69.8 (1.38)	
Registrar	49 (21.3)	67.8 (1.48)	
GRH: Speciality (or speciality in training) present during IO attempt^e^ (*n* = 230)	*n* (%)	First attempt success rate (%)	
Pediatrician	181 (78.7)	70.7	
Anesthesiologist	36 (15.7)	86.1	
Surgeon	5 (2.2)	60.0	
Pediatrician and anesthesiologist	8 (3.5)	50.0	
GRH: hospital unit where attempt occurred^e^ (*n* = 233)	*n* (%)		
ICU	141 (60.5)		
of which:			
pediatric ICU	76 (53.9)		
mixed ICU	49 (34.8)		
neonatal ICU	15 (10.6)		
adult ICU	1 (0.7)		
Emergency department	51 (21.9)		
Pediatric ward	29 (12.5)		
Operating theatre, recovery or imaging	12 (5.2)		
Status and preparation of patient in GRH setting^e^ (*n* = 234)	*n* (%)		
Unconscious	152 (65.0)		
Sedation	40 (17.1)		
Local anesthesia	22 (9.4)		
of which:			
Unconscious & Sedation	19 (8.1)		
Sedation & local anesthesia	9 (3.9)		
Unconscious & local anesthesia	8 (3.4)		
Others	*n* (%)		
Survival to hospital discharge (*n* = 417)	258 (61.9)		
GRH^e^ with specific IO SOP or guideline (*n* = 234)	66 (28.2)		
GRH^e^ personnel with prior training (*n* = 234)	176 (75.2)		

^a^ 1 unknown.

^b^ 17 unknown, 78 post mortem, 15 transferred with IO in place.

^c^ Total includes attempts in which exact site of location was unknown (either left or right missing or unknown whether distal or proximal.

^d^ 1 successful attempt by paramedic or nurse.

^e^ “GRH”: Item was only requested from attempts in a GPSU-reporting hospital (short GRH).

A total of 81.9% of all IO attempts worked at the first attempt, while 98.3% of patients had at least one working access for a long enough period to give necessary drugs (classified as overall success). First attempt success was achieved significantly more often in children older than 1 year of age (*p* = 0.034), in a prehospital setting (*p* = 0.000017) and when first attempts took less than 2 min (*p* = 0.00067). An overview of success rates and complication dynamics by age group can be found in Supplementary Figure S1 Content section.

Almost 3/4 of patients (74.4%) had no complications during their hospitalization related to the establishment of intraosseous access. In 92 patients (22.7%), minor complications, classified as extra or paravasation, misplacement into soft tissue or local swelling, occurred. Ten patients (2.5%) experienced at least one potentially severe complication, including compartment syndrome (*n* = 4), necrosis (*n* = 5), soft tissue infections (*n* = 2), poor perfusion of the foot (*n* = 1) and potential piercing of the tibial bone (*n* = 1). In 0.5% of attempts, technical difficulties caused by insufficient charges in the battery due to old equipment or frequent testing in the equipment check routine were encountered. In children younger than 1 year of age, significantly more complications were encountered (*p* = 0.009).

In terms of indicators of correct placement, out of all attempts with complete information that were successful at the first attempt (*n* = 169), 76.9% had a firm fit in the bone, and 77.5% showed no signs of paravasation. In 53.8% of cases, aspiration of blood/bone marrow was reported. A total of 11.7% of initially successful IO accesses dislocated after some time of use.

The proportions of potentially influencing factors on IO access outcomes are detailed in Table 2. The effects of individual factors are displayed in Supplementary Figure S2–S4 Content section.

**Table 2:** Proportion of potentially influencing factors on IO-access outcomes.

Potentially influencing factors	All children	A. Complications		B. Success at first attempt		C. Dislocation	
		Yes	No	Yes	No	Yes	No
Number of children	417	102	315	102	315	50	367
Age							
<1 year	173 (41.5)	**54** **(31.2)**	**119** **(68.8)**	**130** **(75.1)**	**43** **(24.9)**	21 (12.1)	152 (87.9)
>1 year	244 (58.5)	**48** **(19.7)**	**196** **(80.3)**	**205** **(84.0)**	**39** **(16.0)**	29 (12.1)	215 (88.1)
Access setting							
GRH setting	219 (52.5)	61 (27.9)	158 (72.1)	**158** **(72.1)**	**61** **(27.9)**	21 (9.6)	198 (90.4)
Prehospital/non-GRH setting	198 (47.5)	41 (20.7)	157 (79.3)	**177** **(89.4)**	**21** **(10.6)**	29 (14.6)	169 (85.4)
Duration of first IO access							
>2 min	127 (55.7)	**48** **(37.8)**	**79** **(62.2)**	**80** **(63.0)**	**47** **(37.0)**	16 (12.6)	111 (87.4)
<2 min	101 (44.3)	**20** **(19.8)**	**81** **(80.2)**	**85** **(84.2)**	**16** **(15.8)**	12 (11.9)	89 (88.1)
Doctor's seniority							
Consultant/Department head	119 (51.1)	40 (33.6)	79 (66.4)	81 (68.1)	38 (31.9)	15 (12.6)	104 (87.4)
Other personnel^a^	114 (48.9)	33 (28.9)	81 (71.1)	86 (75.4)	28 (24.6)	16 (14.0)	98 (86.0)
Doctor's speciality							
Non-pediatrics	49 (21.1)	12 (24.5)	37 (75.5)	38 (77.6)	11 (22.4)	3 (6.1)	46 (93.9)
Pediatrics	183 (78.9)	61 (33.4)	122 (66.7)	129 (70.5)	54 (29.5)	28 (15.3)	155 (84.7)
CPR							
Yes	199 (47.7)	50 (25.1)	149 (74.9)	163 (81.9)	36 (18.1)	25 (12.6)	174 (87.4)
No	218 (52.3)	52 (23.9)	166 (76.1)	172 (78.9)	46 (21.1)	25 (11.5)	193 (88.5)
Consciousness status							
Conscious	262 (62.8)	**55** **(21.0)**	**207** **(79.0)**	218 (83.2)	44 (16.8)	30 (11.5)	232 (88.5)
Unconscious	155 (37.2)	**47** **(30.3)**	**108** **(69.7)**	117 (75.5)	38 (24.5)	20 (12.9)	135 (87.1)

Values shown are *n* (%). Bold text presents significant differences.

^a^ Others include registrar, specialist, and nurse/paramedic.

The success or failure of an IO attempt in our data does not significantly depend on which system was used (*p* = 0.2096).

Analyzing the effect of individual factors on the occurrence of moderate and severe patient-side complications, a univariate model identified unconsciousness, age <1 year and prolonged insertion times (>2 min) as proxies for a difficult placement setting. In a multivariate model using stepwise backward selection, a prolonged insertion time (OR 2.58) and prehospital/non-GRH (GPSU-reporting Hospital) setting (OR 9.29) significantly increased the occurrence of complications. We further investigated the factors that influenced first-time success. The multivariate backward selection model identified only a time >2 min to first working access as a significant positive influencing factor on first attempt success. In a similar fashion, the effect of a set of factors on dislocation was assessed. A highly significant OR of 17.74 was identified for dislocations occurring in a prehospital/non-GRH setting.

Additional data on diagnoses leading to intraosseous access attempts, medications applied via the intraosseous route as well as success rates and complication occurrence by age group can be found in Tables 3–5 respectively.

**Table 3:** Diagnoses leading to IO access in children aged 1 month to adulthood.

Diagnosis (indication for IO)	Total population (*n* = 417)	Of which with CPR
Resuscitation (CPR)	134 (32.1)^b^	134 (100.0)
Seizure/status epilepticus	71 (17.0)	5 (7.0)
Shock/sepsis	55 (13.2)	12 (21.8)
Respiratory insufficiency	47 (11.3)	16 (34.0)
Trauma	19 (4.6)	7 (36.8)
Congenital heart defect	18 (4.3)	11 (61.1)
Respiratory insufficiency & shock/sepsis	18 (4.3)	–
Drowning	7 (1.7)	6 (85.7)
Thermal injury	9 (2.2)	–
Diabetic ketoacidosis	7 (1.7)	–
Bolus event/aspiration	6 (1.4)	4 (66.7)
Access needed for operative intervention	6 (1.4)	–
Arrhythmia	5 (1.2)	4 (80.0)
Coma/loss of consciousness	5 (1.2)	–
Dehydration/exsiccosis	3 (0.7)	–
Anaphylaxis	2 (0.5)	–
Other^a^	5 (1.2)	–

Values presented are *n* (%).

^a^ One case each of hypothermia, metabolic imbalance, poor peripheral vein status, SIDS, acute-onset abdominal pain.

^b^ Of which 2 (1.5%) received an additional ECMO-Intervention. In 199 cases the indication included ongoing cardio-pulmonary-resuscitation (CPR).

**Table 4:** Medications applied in study patients via the intraosseous access route.

Medications	Number of patients receiving the medication (*n* = 417)	% of patients receiving medication (*n* = 417)
Cristalloids	342	82.0%
Adrenaline	177	42.4%
Sedation	107	25.7%
Analgesics	72	17.3%
Antiepileptics/anticonvulsives	48	11.5%
Induction of anaesthesia	40	9.6%
Antibiotics	20	4.8%
Blood products	21	5.0%
NaBic	19	4.6%
Colloids	19	4.6%
Vasoactive therapy (Akrinor, Noradrenaline, Dobutamine)	15	3.6%
Prednisolon/cortison	11	2.6%
Others	58	13.9%

**Table 5:** Success rates and complication occurrence by age group.

	0–28 days^b^	1–12 months	1–6 years	6–12 years	>12 years	1 month–>12 years
Overall success rate	90.7	97.7	97.9	100.0	100.0	98.3
Success at first try	74.7	69.9	72.0	90.0	81.0	72.9
Overall rate of complications^a^ (minor & potentially severe, excl. technical)	**29** **.** **6**	**31** **.** **8**	**23** **.** **4**	**7** **.** **4**	**10** **.** **0**	**25** **.** **2**
Rate of severe complications	6.2	4.7	1.1	0.0	0.0	2.5

Values shown are %.

^a^ Significant at the 5% level.

^b^ Data from Schwindt et al. (2022).

## Discussion

To our knowledge, this dataset of systematically documented intraosseous access attempts constitutes one of the largest published in the literature.

Our study found that both the chance of success at the first attempt and the overall success rate increased significantly with the age of the child. In parallel, the overall rate and rate of severe complications decreased sharply as the size and age of the child increased, which were consistent with Capobianco et al.'s (9) study in that a higher risk of incorrect positioning was found in smaller target structures. Schwindt et al. (10) found that this trend continues in patients receiving an IO in their first 24 h of life, with higher gestational age increasing the overall success rate.

By comparing the success rates in our study with those in other studies, we found that the success rate for all attempts in our cohort was higher at 81.2% than the 65.0% success rate in the cohort of 4,270 pediatric patients in the study by Rosetti et al. (11) and that the mean number of attempts in our cohort was lower at 1.31 vs. 1.54 and the EZIO-specific (the product name of the most frequently-used intraosseous access drill by the company Arrow) success rate in our cohort was higher at 81.2% vs. 70.0% (12). Myers et al. reported the results of 62 patients, which are in line with the findings of our study, with a first attempt success rate for EZIO devices of 83.9% vs. 81.9% (12). Sunde et al. published a 96% overall success rate for the EZIO system, which is higher than the 82.0% (*n* = 460) achieved in this cohort, while 55% for the Cook needle is below our estimate of 68.0% (*n* = 25) for Cook needles (13). Helm et al. (14) even found a 100% overall and 87.5% first try success rate in children smaller 7 years of age in a prehospital setting. Our data suggests a significant difference in first attempt success rate between children smaller 1 year of age and children older 1 year of age of 75.1% and 84.0% respectively.

Individual cases of manual EZIO placement were reported in children less than 12 months of age. This represents a possible alternative for placement in small children, as also shown for the neonatal cohort by Schwindt et al. (10).

The proximal tibia is the preferred location for IO access due to its ease of identification, thin layer of subcutaneous tissue and relatively large target area, which is well reflected in our data.

Somewhat counterintuitively, prolonged placement of the first IO access increases the risk for complications by 2.58 and the rate for success on the 1st attempt by 3.3. We attribute these increases to prolonged placement and preparation (e.g., time spent for optimal positioning).

According to Leidel et al., the first-time success rate of IO access during resuscitation in a level 1 trauma center in adults was 85% and that of central venous catheter (CVC) placement was 60% (15).

A very early study from Rosetti et al. (16) in 1985 found a very low rate of potentially severe complications of only 0.9%, of which 0.6% were osteomyelitis. The low rate of potentially severe complications in our cohort was 2.5%, which constitutes a justifiable risk in terms of the urgent need for quick vascular access in life-threatening emergencies. No cases of osteomyelitis were observed in our cohort. The relatively high rate of minor complications of 22.1% seems acceptable in life-threatening emergency situations and, if detected early, usually does not lead to long-term complications. Mori et al. found a comparable rate of minor complications of 21.6% (17 extravasations of fluid and 4 dermal abrasions) in a pediatric emergency department cohort (17). In the few articles on long-term complications, there are no relevant long-term effects of intraosseous infusion on tibial bone growth (18).

Our study found a strong difference in the rate of complications between children younger and children older than 1 year of age. While the overall rate of complications is 19.7% above 1 year of age, IO access attempts in children younger than 1 year of age encounter complications in almost every third patient (31.2%).

The significantly decreased occurrence of complications and lower failure rate of IO attempts in a prehospital setting might be due to underreporting of additional attempts which were unknown to the admitting center and/or complications at the handover or might be due to prehospital staff being more familiar and experienced with IO devices.

Initially, successfully placed needles dislocated at a relatively high rate of 11.7%, often leading to a necessary second attempt. According to user comments, this was often due to poor fixation in children, although dislocation through high intramedullary pressure by fluid bolus application or other causes might be possible. A sufficient amount of time is needed to precisely fix the cannula, especially in a prehospital environment where dislocations were observed in almost every 7th patient and almost twice as frequently compared to an intrahospital setting (13.3% vs. 7.4%).

Almost two-thirds of patients (61.6%) received alternative access attempts before IO access was attempted. In 248 patients a peripheral venous line, in 21 patients a central venous line and in 1 patient a port puncture was attempted. In the prehospital setting, the percentage of patients receiving alternative access attempts across all indications was 44.9%. Only 47.1% (97/206) of patients with indications for resuscitation in the prehospital setting and in a GPSU-reporting hospital (short GRH) underwent an alternative access attempt before IO access was attempted. In 9 cases, an IO was attempted with an alternative access already in place due to the need for a higher inflow of drugs and/or a further vascular access. According to the ERC guidelines at that time, IO access should be attempted 60 s after attempts to create intravenous access were unsuccessful; thus, because of the low rate of successful IV placement in critically ill children, IO access should be the primary access method (4, 7). Lee et al. (19), however, discovered that providers might still be hesitant to use IO in non-CPR settings.

Interestingly, frequent use of IO accesses during seizures (*n* = 66, 15.8%) as a means of infusing anti-epileptic/convulsive drugs was reported. This is surprising due to the existence of a less invasive application method, such as intranasal, buccal or intramuscular routes of antiepileptic drug application. Bhattacharyya et al. (20) identified a mean time from arrival at the emergency to the administration of intranasal midazolam of 50.6 +/− 14.1 s, which was shorter than the average IO insertion time by almost 2 min, while Mahmoudian (21) found no significant side effects of intranasal drug application. Another alternative form is intramuscular application of midazolam, which could help to prevent the need for IO access (22).

With almost two-thirds (64.8%) of all IO needles being removed within 12 h of placement and only a small fraction (3.0%) remaining in place for more than 24 h, our data confirm that intraosseous access is mainly used to establish quick emergency access to bridge emergency situations until a more permanent access, such as a central or peripheral venous catheter, can be established. This finding is also in accordance with guidelines (7) in which the maximum use time is 24 h and the ideal removal time is 2 h after arrival at the hospital and replacement with another access method. Suominen et al. (23) suggests aiming for early removal of the IO needle and ensuring vigilant and frequent monitoring of perfusion in the affected extremity. In contrast, Philbeck et al. (24) demonstrated that it might be safe to maintain the EZIO in place for up to 48 h in an adult cohort. The German guideline on intraosseous infusion, however, recommends interpreting these results with great care and only as preliminary findings (7).

Pediatricians and anesthesiologists performed 97.9% of all access attempts in a GRH setting, with a board qualified physician being present in 78.4% of cases and a consultant or department head in 51.1% of cases. Intensified training efforts on IO indication, handling and placement would be well advised for those two specialties, and more generally, all medical personnel, doctors, nurses and paramedics treating children (25) should have timely access to and be familiar with and well trained in the use of intraosseous drills and access methods (7).

Regular training is important and leads to high success rates, as demonstrated by a simulation-based IO-training study using manikins. Thus, it is reasonable to conclude that six months after a single training session, the success rates for correct identification of the puncture site as well as successful puncture at the first attempt are high (26, 27).

## Limitations

Multiple limitations apply to our data. Data being acquired via German hospitals only might limit the applicability of the data in settings where training, SOPs, and operating circumstances might differ widely. Evaluating details of an emergency situation, often filling out the questionnaire sometime after the event, might have led to misinformation or lost details. Time to establish the io access was estimated, not measured. Reconstructing the chain of events and their success rate from a retrospective questionnaire posed some significant challenges that might have also introduced some discrepancies in how events truly occurred. GPSU might have introduced a significant underreporting bias. Children who did not arrive at a GRH, for example, because resuscitation was discontinued in a prehospital setting, were not reported to GPSU and are not included in our data. The lack to follow-up this population might introduce a significant bias as there might potentially be significant discrepancies in malpositioning rates in the post-mortem cohort as suggested by Maxien et al. (28) There is a possibility of an underreporting bias of critical incidence, as reporting was voluntary. There was no further information on the extent or area of the complications available.

## Conclusion

This study supports the recommendations of international guidelines regarding intraosseous access use. First attempt and overall success increase significantly with the age of the child, while the rate of minor and severe complications decrease significantly in children older one year of age. Given the paramount need for quick vascular access in emergency situations the rate of severe complications is low, while the chance of achieving successful emergency access is high. Dislocations occur in almost 1 out of 10 cases and twice as frequently in the prehospital environment. We conclude that intraosseous access is a frequently used and reliable method for the establishment of vascular access in an emergency situation.

## Data Availability

The raw data supporting the conclusions of this article will be made available by the authors, without undue reservation upon reasonable request.
