# Supplementary material for: Factors influencing the success and complications of intraosseous access in pediatric patients—a prospective nationwide surveillance study in Germany
Source: Front Pediatr. 2023 Nov 29;11:1294322. doi: 10.3389/fped.2023.1294322 (PMC10716217; doi:10.3389/fped.2023.1294322)
Supplement: Supplementary file 2 [file Datasheet1.pdf]

Hospital Questionnaire

(translated from the original German version)

Filled out by GPSU:
Date of birth: ____ /_______ Sex: female / male Zip-Code Residence: ______XX

1. Patient data

Week of Gestation (for Neonates <28 days): . . . .

Age of patient at time of IO attempt:

☐ 0-24 h ☐ 1-7 days ☐ 8–28 days ☐ 1-12 months ☐ 1-6 years ☐ >6 -12 years ☐ >12 years

2. Did the patient arrive with an IO access at your hospital?

☐ **yes** *if yes*: How many? .... ☐ 1 ☐ 2 ☐ >2
Did the IO access work on admission? ☐ yes ☐ no ☐ unknown ☐ was no longer needed

☐ **no** *if no*: ➨ *continue with section 3.*

The following questions refer exclusively to the IO access placed prehospitally / in the external hospital:

Which system was used? *(Multiple answers possible)*

☐ Cook-Needle ☐ Cook-Needle Imitation ☐ EZIO-Drill
☐ Bone Injection Gun (BIG) ☐ Other(s): . . . .

Which puncture site(s) were used? *(Multiple answers possible)*

☐ proximal tibia (☐ right / ☐ left) ☐ distal tibia (☐ right / ☐ left) ☐ distal femur (☐ right / ☐ left)
☐ proximal humerus (☐ right / ☐ left) ☐ other(s): . . . .

Were alternative access routes such as peripheral venous access (IV) or umbilical venous catheter (UVC) attempted prior to placement of the IO access?

☐ yes ☐ no ☐ unknown
if yes, which? ☐ IV ☐ with / ☐ without success
☐ UVC ☐ with / ☐ without success

Which substances / drugs were applied via IO access? *(Multiple answers possible)*

☐ Cristalloids ☐ Colloidal volume ☐ Adrenaline ☐ Amiodarone ☐ Adenosine
☐ Analgesics ☐ Antiepileptic drugs ☐ Sedation ☐ Anesthesia induction ☐ Blood products
☐ Other(s): . . . .

What complications and problems occurred? *(Multiple answers possible)*

☐ none
☐ Misplacement into soft tissue ☐ Soft tissue infection ☐ Fat embolism
☐ Extra-/Paravasation ☐ Osteomyelitis ☐ Necrosis
☐ Compartment-Syndrome ☐ Fracture ☐ Other(s): . . . .

After what time was/were the IO access(es) removed?

☐ <1 h ☐ 1-6 h ☐ 6-12 h ☐12-24 h ☐ >24 h ☐ post mortem

3. Indication for IO access

Due to which disease did the child receive an IO access?

☐ Respiratory insufficiency ☐ Reanimation ☐ Perinatal Asphyxia ☐ Shock/Sepsis ☐ Trauma
☐ Cardiac arrhythmia ☐ Intoxication ☐ Congenital heart defect ☐ Seizure ☐ Anaphylaxia
☐ Thermal injury ☐ Other(s): . . . .

4. Vital signs on admission to the hospital or at the time of decision for IO access inhospital

Ongoing CPR? ☐ yes ☐ no
GCS: ☐ unknown ☐ 3 ☐ 4-8 ☐ 9-12 ☐ 13-15
HR/min: ☐ unknown ______ /min ☐ Asystole ☐ Ventricular fibrillation
RR/min: ☐ unknown ______ /min ☐ Apnea
BP syst / diast (MAD): ☐ unknown _____ / _____ (_____) ☐ not measurable
CRT: ☐ unknown ☐ <2 sec. ☐ 3 sec. ☐ 4 sec. ☐ 5 sec. ☐>6
Ventilation:
☐ yes *if yes*: ☐ Invasive via tube ☐ Laryngeal mask ☐ Laryngeal tube
☐ no ☐ Non-invasive ventilation (NIV) ☐ CPAP

For newborns: Apgar-Score: ___ / ___ / ___ (1 / 5 / 10 min.) ☐ unknown

All further questions refer exclusively to the IO accesses placed in your hospital:

5. Did the patient receive an IO access at your hospital?

☐ **no** *if no:* ➨ continue with section 6.

☐ **yes** *if yes:* ➨ continue here ⬇

How many IO accesses did this patient receive in your clinic during initial treatment?
☐ 1 ☐ 2 ☐ 3 ☐ 4 ☐ >4

Where was the IO access(es) placed in your hospital? *(Multiple answers possible)*
☐ Emergency room ☐ Children ward ☐ NICU ☐ Mixed ICU (NICU + PICU)
☐ OP ☐ Infant ward ☐ PICU ☐ Delivery room
☐ Other(s): . . . .

Who placed the IO access(es) in your hospital? *(Multiple answers possible)*
☐ Pediatric Resident ☐ Pediatric Specialist ☐ Pediatric Consultant ☐ Nurse
☐ Anesthesiology Resident ☐ Anesthesiology Specialist ☐ Anesthesiology Consultant
☐ Other(s): . . . .

Which system was used? *(Multiple answers possible)*
☐ Cook-Needle ☐ Cook-Needle Imitation ☐ EZIO-Drill ☐ Bone Injection Gun (BIG)
☐ Other(s): . . . .

Which puncture site(s) were used? *(Multiple answers possible)*
☐ proximal tibia (☐ right / ☐ left) ☐ distal tibia (☐ right / ☐ left)
☐ proximal humerus (☐ right / ☐ left) ☐ distal femur (☐ right / ☐ left)
☐ other(s): . . . .

How many puncture attempts were needed before the first IO needle was correctly positioned?
☐ 1 ☐ 2 ☐ 3 ☐ 4 ☐ 5 ☐ 6 ☐ >6

If >1 puncture attempt: what were the problems with placing the IO access? (Item in red was added after revision of the questionnaire during the course of the study.)

. . . .

What is the estimated time from indication to successful placement of the first IO access?

☐ <1 min ☐ <2 min ☐ <3 min ☐ <4 min ☐ <5 min ☐ >6 min

How was the correct position of the cannula verified? *(Multiple answers possible)*

☐ Bone marrow aspiration
☐ Firm hold of the needle after removal of the trocar
☐ No extravasation with injection of a volume bolus

Was the patient unconscious at the time the IO needle was inserted? ☐ yes ☐ no
Was analgesia used to establish the IO line? ☐ yes ☐ no
Were local anesthetics used to reduce infusion pain? ☐ yes ☐ no

Which substances / drugs were applied via IO access? *(Multiple answers possible)*

☐ Cristalloids ☐ Colloidal volume ☐ Adrenaline ☐ Amiodarone ☐ Adenosine
☐ Analgesics ☐ Antiepileptic drugs ☐ Sedation ☐ Anesthesia induction ☐ Blood products
☐ Other(s): . . . .

What complications and problems were encountered in the course of establishing IO access? *(Multiple answers possible)*

☐ none ☐ Selection of wrong needle length ☐ Misplacement into soft tissue ☐ Extra-/Paravasation
☐ Compartment-Syndrome ☐ Soft tissue infection ☐ Osteomyelitis
☐ Fracture ☐ Fat embolism ☐ Necrosis
☐ Other(s): . . . .

After what time was/were the IO access(es) removed?

☐ <1 h ☐ 1-6 h ☐ 6-12 h ☐12-24 h ☐ >24 h ☐ post mortem

Were alternative access routes such as peripheral venous access (IV), umbilical venous catheter (UVC) or central venous catheter (CVC) attempted prior to placement of the IO access?

☐ yes *if yes, which?* ☐ IV ☐ with / ☐ without success How many attempts? ___
☐ no ☐ UVC ☐ with / ☐ without success How many attempts? ___
☐ CVC ☐ with / ☐ without success How many attempts? ___

Does your hospital have a standardised guideline or SOP for the use of IO access? ☐ yes ☐ no
Are all persons who use the EZIO trained in its use? ☐ yes ☐ no

6. Course and Outcome

What other measures were performed during the initial care within the first two 2 hours? *(Multiple answers possible)*

☐ none ☐ Mask ventilation ☐ Intubation + Ventilation ☐ Alternative airway management (LMA, LT)
☐ Chest drainage ☐ Defibrillation ☐ CPR with chest compressions
☐ CVC placement ☐ Arterial line placement ☐ Catecholamine therapy
☐ UVC placement

Did the patient survive? ☐ yes ☐ no

As a user, how satisfied were you with the use of IO access?

☐ very satisfied ☐ satisfied ☐ rather not satisfied ☐ dissatisfied ☐ very dissatisfied

What should be improved in your opinion? . . . .

. . . .

7. Additions / Comments *(If necessary, also use the back side)*

Please return the completed questionnaire to the GPSU office!
THANK YOU FOR YOUR COOPERATION!
