# Supplementary figures and images for: Factors influencing the success and complications of intraosseous access in pediatric patients—a prospective nationwide surveillance study in Germany
Source: Front Pediatr. 2023 Nov 29;11:1294322. doi: 10.3389/fped.2023.1294322 (PMC10716217; doi:10.3389/fped.2023.1294322)

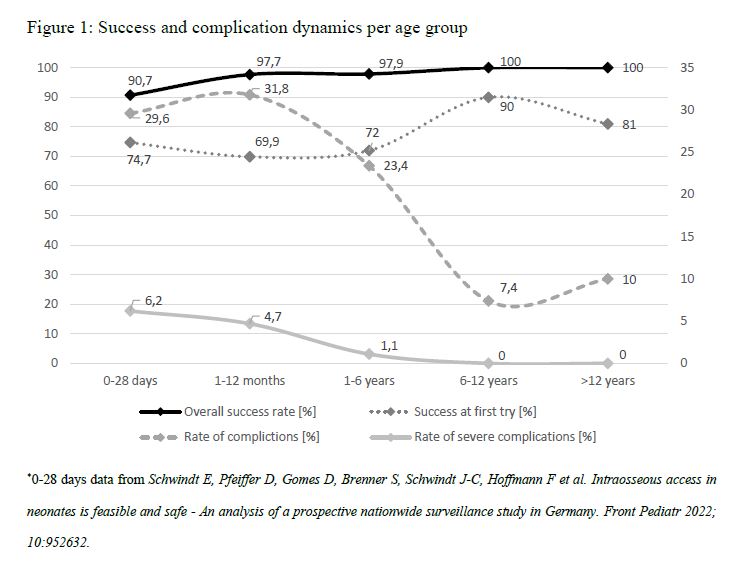

Supplement: Supplementary file 3 [file Image1.jpeg]

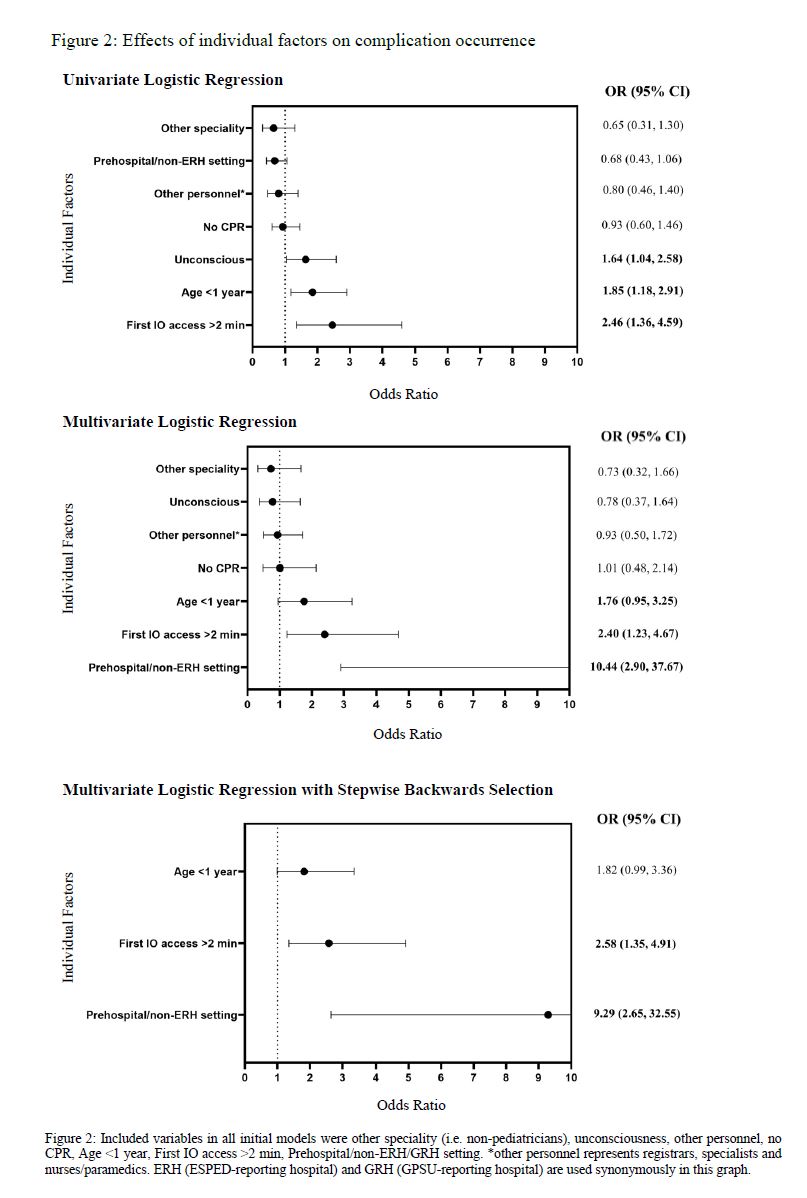

Supplement: Supplementary file 4 [file Image2.jpeg]

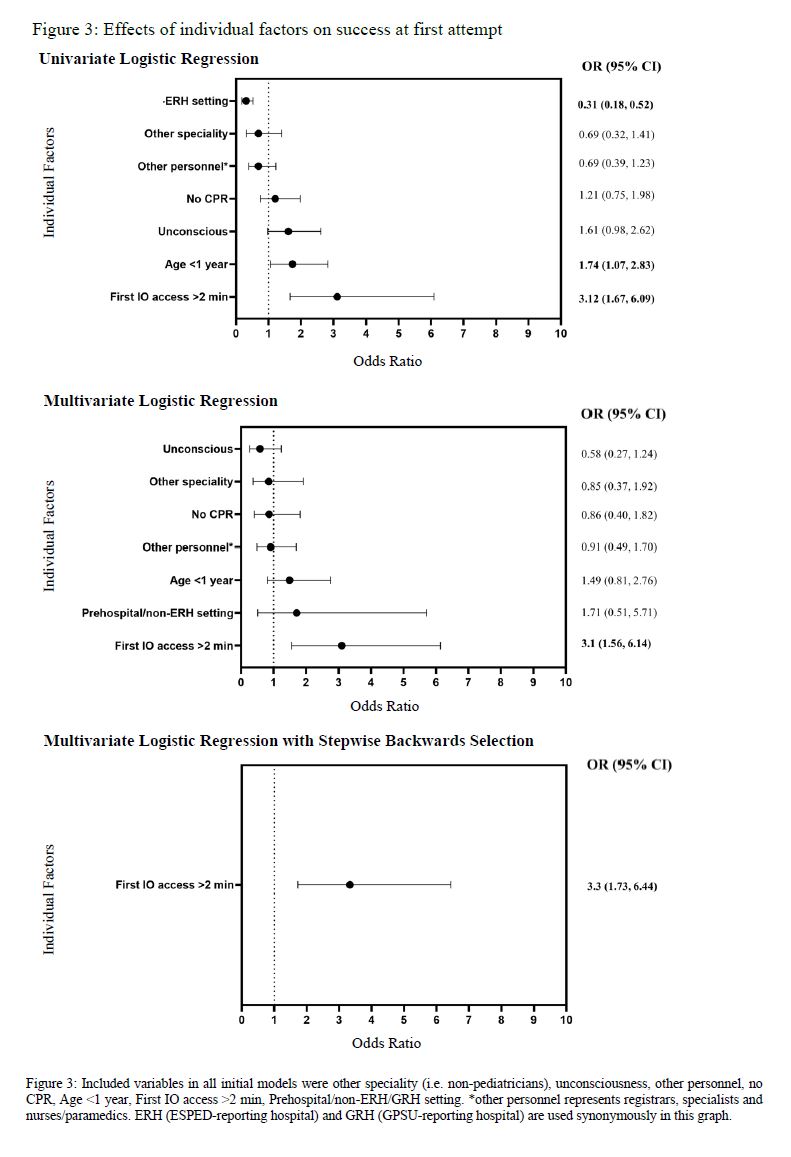

Supplement: Supplementary file 5 [file Image3.jpeg]

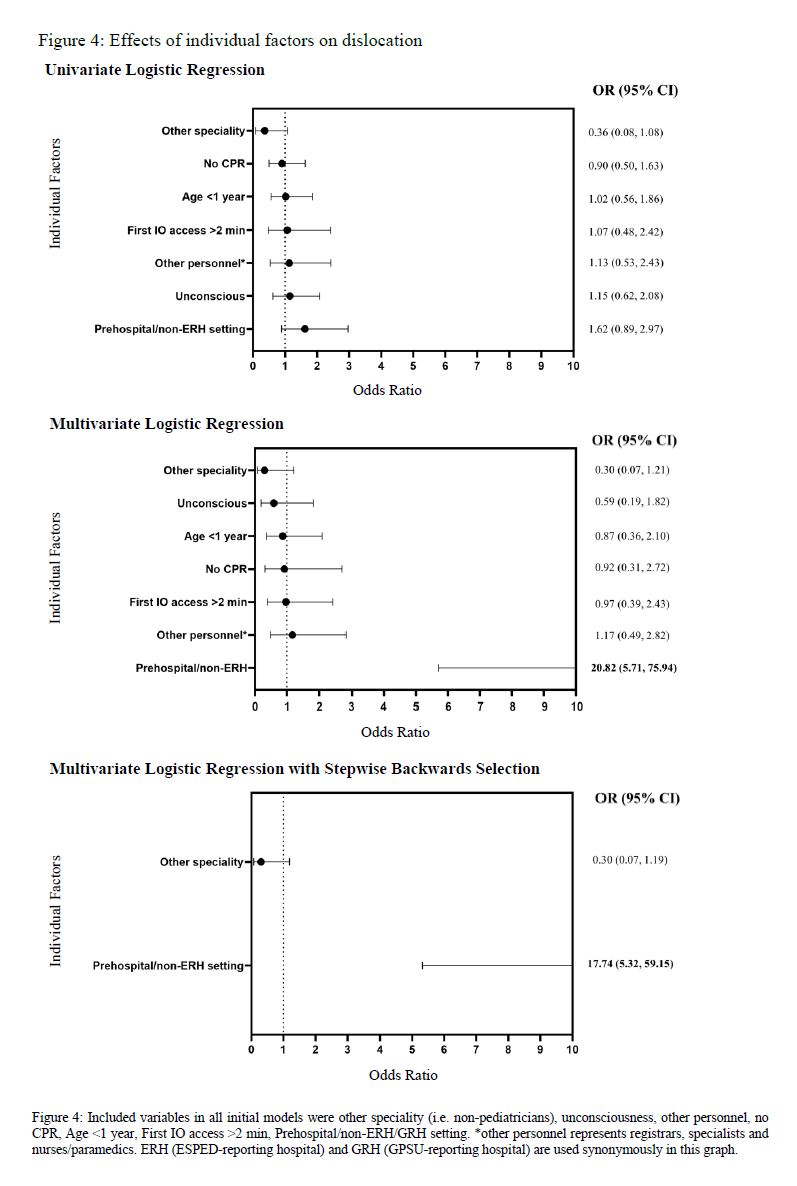

Supplement: Supplementary file 6 [file Image4.jpeg]
